# Development and validation of an informative manual on venous thromboembolism for the lay population

**DOI:** 10.31744/einstein_journal/2020AO5425

**Published:** 2020-09-09

**Authors:** Nadya Cerqueira Takara, Natany da Costa Ferreira, Beatriz Murata Murakami, Camila Takao Lopes

**Affiliations:** 1 Universidade Federal de São Paulo São PauloSP Brazil Universidade Federal de São Paulo, São Paulo, SP, Brazil.; 2 Universidade de São Paulo São PauloSP Brazil Universidade de São Paulo, São Paulo, SP, Brazil.; 3 Hospital Israelita Albert Einstein São PauloSP Brazil Hospital Israelita Albert Einstein, São Paulo, SP, Brazil.

**Keywords:** Venous thromboembolism, Risk factors, Education, nursing, Pulmonary embolism, Validation study

## Abstract

**Objective:**

To develop an informative manual on venous thromboembolism prevention for the lay population and to estimate evidences of content and face validity.

**Methods:**

A methodological study conducted in three stages. The first stage was the preparation of the manual, followed by content validation with cardiovascular specialists who judged clarity, theoretical relevance and practical pertinence on a 4-point Likert scale. Items with a content validity index ≤0.75 were revised and re-evaluated. The last stage was the face validation by lay people, who were interviewed regarding item understanding and visual appearance. Items with more than 80.0% positive opinions were considered adequate.

**Results:**

The manual was developed containing nine illustrations, definition of the disease, risk factors, signs and symptoms, and preventive measures. In the first assessment round, the validity index was 1.0 for the text of all sections, with suggestions for language adjustments. As to the illustrations, the validity indexes ranged from 0.67 to 1.0. In the second round, the validity index reached 1.0 for all items. A total of 40 lay people participated in the face validation, and all considered the paper type and font size appropriate, as well as the font used as readable; 97.5% were able to understand the information contained in the manual; 98.0% considered it esthetically beautiful; and 90.0% considered the reading not tiresome.

**Conclusion:**

The informative manual on venous thromboembolism prevention was prepared, its content validated by experts, and considered appropriate by the lay population. These results suggest that the manual may be used as a preventive educational strategy for venous thromboembolism.

## INTRODUCTION

Venous thromboembolism (VTE) includes deep vein thrombosis (DVT) and pulmonary thromboembolism (PTE) and ranks third as major cardiovascular disease (CVD). The disease is characterized by formation of thrombi in deep veins, mainly in the lower limbs, causing pain, edema, warmth, tenderness and ulcers. Thrombi can detach from the vessel walls and travel through the circulation, obstructing the pulmonary vessels.^(1-4)^

Approximately 10 million people in the world are affected by the disease.^(2)^ More than 500 thousand deaths in Europe and over 300 thousand in the United States occur annually due to VTE.^(5)^ In Brazil, although it remains high, the crude mortality rate due to PTE decreased by 31%, in a progressive and steady way, from 2.8/100 thousand in 1989 to 2.62/100 thousand in 2010.^(6)^

From July 2018 to July 2019, the Brazilian Public Health System (SUS - *Sistema Único de Saúde*) spent approximately R$ 29 million on more than 47 thousand hospital admissions due to VTE, with a mortality rate of 2.46%.^(7)^These costs are due to the treatment of VTE, including chronic secondary prevention for 3 to 6 months: anticoagulation with unfractionated heparin, low molecular weight heparin, new oral anticoagulants and vitamin K antagonists.^(8)^ For patients with two or more spontaneous thrombotic episodes, anticoagulant therapy should be carried out indefinitely.^(9)^

Despite the high mortality rate associated with the disease, lay people know little about VTE, as compared to knowledge about symptoms and risk factors of other CVD.^(10)^ As pointed out in a survey carried out by the Brazilian Institute of Public Opinion and Statistics (IBOPE - *Instituto Brasileiro de Opinião Pública e Estatística*) in 2010, the Brazilian lay population is aware about the existence of this disease, but does not know how to treat, prevent or control it.^(11)^

Risk factors for VTE include age, sex, ethnicity, body mass index/obesity, use of oral contraceptives or hormone therapy, use of steroids, sedentary habits, cancer, surgeries, trauma/fractures, immobilization, pregnancy/puerperium, long trips and acute infections, among others.^(12)^ The fact that the lay population ignores such risk factors and their preventive measures, is a hindrance to prevention. Therefore, educational strategies for the lay population must be developed in order to avoid the development of the disease and hospitalizations, and reduce the costs associated with treatment. Verbal information is very dense, therefore written materials help patients remember care they must take, and the can be consulted even when the healthcare professional is not present.^(13)^

One aspect to be considered when using printed educational material is validity of this instrument, which refers to confirming that its use meets the purpose of its development.^(14)^ Previous studies have developed and validated written educational materials to guide patients in different contexts.

Cruz et al.,^(15)^ developed a manual for head and neck cancer patients who underwent radiation therapy, after searching scientific articles and technical books, and validated the content through the opinion of expert professionals.^(15)^ Other studies also considered the opinion of the target public. Lopes et al.,^(16)^ developed an information manual on bed bathing for coronary artery disease patients, which was later validated by both nurses and patients.^(16)^ The same group later developed an information manual on cardiac catheterization, also validated by both groups.^(17)^ Oliveira et al.,^(18)^ prepared a booklet on healthy eating for pregnant women, which was validated by specialist professionals and pregnant women.

To the best of our knowledge, written educational materials for lay people regarding prevention of VTE have not been prepared and validated. The development of such materials will fill this knowledge gap.

## OBJECTIVE

To prepare an information guide on prevention of venous thromboembolism for the lay population and estimate the evidences of content and face validity.

## METHODS

A methodological study carried out in three stages: preparation of the manual, content validation by experts, and face validation by lay people.

### First stage: preparation of the manual

The manual was prepared in 2018, by a third-year undergraduate nursing student, under the supervision of a professor, a nurse specialized in cardiology, with a doctorate degree.

The texts were based on scientific articles published on PubMed^®^ and Scientific Electronic Library Online (SciELO) databases, from 2014 to 2018.^(19-23)^ The non-systematized search was performed using the terms “*tromboembolismo venoso*” and “venous thromboembolism”, in the fields “title or abstract”. Studies addressing the definition of the disease, signs and symptoms, risk factors and preventive measures were selected.

Information was extracted from studies by the undergraduate student and reviewed by the professor, and arranged in the initial version of the manual aiming to address literate lay people, with minimum schooling level of incomplete primary education. The manual was printed on A4 90g/mm paper. The illustrations and the folder format were created by a graphic designer.

The preparation followed the recommendations by Hoffman et al.,^(13)^ for development of effective written health educational materials: involve the main stakeholders including patients; clearly state the purpose of the material; focus on providing information that is behavior-focused (*e.g*., “it is important that you do the exercises every day”); ensure that the content is accurate, up-to-date, evidence-based, and sources appropriately referenced; include the authors’ names on the material and the publication date; avoid judgmental or patronizing language; Aim for fifth to sixth grade reading level; use short sentences, expressing only one idea per sentence; use common words wherever possible; avoid the use of jargon or abbreviations; write in the active voice and in a conversational style; write in the second person (*e.g*.: “you” rather than “the patient”); Sequence the information so that the information that patients most want to know is at the beginning; use subheadings; present the information using bulleted lists whenever possible; use a minimum 12 point font size; avoid the use of italics and all capitals; only use bold type to emphasize keywords or phrases; ensure good contrast between the font color (*e.g*.: black) and the background (*e.g*.: white); only use illustrations if they will enhance the reader’s understanding; use simple-line drawings that are likely to be familiar to the reader.

### Second stage: content validation

The informative manual was evaluated by a non-probabilistic, convenience sample, consisting of nurses specialized in the cardiovascular field, with minimum clinical experience of 2 years, plus experience in the areas of teaching and research, all participants in a study, research and assistance group from a federal university.

The sample size was determined according to the recommendations by Cassepp-Borges et al.,^(14)^ considering six specialists as the minimum acceptable number for this type of study. The experts were invited to participate in the survey by email. Those who agreed to participate received a questionnaire for demographic and professional characterization, one for the evaluation of the manual content, and the Informed Consent Form (ICF). The characterization variables for the experts were sex, age, higher level of education, years of clinical, teaching, and research experience in the cardiovascular field.

The experts individually analyzed each item of the manual, using the following psychometric criteria:^(24,25)^ clarity (the item was written in such a way that the concept was understandable and adequately expressed what was expected to be measured); theoretical relevance (the item reflected the concepts involved, it was relevant and adequate to achieve the proposed objectives); practical appropriateness (the item reflected the concepts involved, it was relevant and adequate to achieve the proposed objectives). The response magnitudes, for all criteria, were 1 for not clear/relevant/appropriate, 2 for a little clear/relevant/ appropriate, 3 for clear/relevant/appropriate enough, 4 for very clear/relevant/appropriate.

In addition to the psychometric criteria, suggestions were asked in order to improve the manual when the assessment was different from “4”. The experts were given 15 days to electronically return the completed questionnaires. In case of non-compliance with the initial return deadline, a new electronic contact was made, giving another 10-day window for return.

The content validity index (CVI) is the proportion of items scored 3 or 4 by the experts and was calculated for each item. Items with CVI ≤0.75 were sent back to the experts for a new evaluation, until the acceptable CVI was reached.^(24,25)^

The data were stored, organized and analyzed in a Microsoft Office Excel spreadsheet. The characterization variables of the experts were described by measures of central tendency and dispersion. For categorical variables, absolute and relative frequencies were calculated; for quantitative variables, mean, standard deviation, minimum and maximum values were calculated.

### Third step: face validation

The informative manual was judged by a non-probabilistic, convenience sample, comprising people without any professional experience in the field of health, aged 18 years or older, of both sexes.

Recruitment was carried out by the undergraduate student in a gated community condo, when the objectives of the study and the form of participation of the subjects were explained. Those who agreed to participate signed the ICF, read the manual, and answered a questionnaire on demographic characteristics. The characterization variables were age, sex, and level of education.

After filling in demographic data, the tool to evaluate the manual was applied through a structured interview, with dichotomous questions, conducted by the undergraduate student who prepared the manual. Understandability of information in the manual, information load, and esthetic aspects, such as adequacy of size, type of font and type of paper used for printing were evaluated. Items with positive opinions for a proportion equal to or greater than 80% of the sample were considered adequate.^(24,25)^

The project was submitted to and approved by the Internal Review Board of the *Universidade Federal de São Paulo* (CAAE: 93294718.2.0000.5505, protocol 2,795,768). Experts and lay people were guaranteed confidentiality and anonymity.

## RESULTS

The manual was initially prepared with the sections “What is thrombosis?”, “What are the risk factors for thrombosis?”, “Warning signs of thrombosis”, and “How to prevent thrombosis?”.

For content validation, ten experts were invited, and seven (70.0%) agreed to participate in the study. Six (85.7%) returned the completed instruments.

All experts were female (6; 100%) and the mean age was 30.3 (±3.3) years. Most held a master’s degree (4; 66.6%); 6.3 (±2.4) years of clinical experience; 6.8 (±3.5) years of teaching experience, and 2.2 (±4.4) years of research experience in the cardiovascular field.

In the first round of content validation, only two illustrations (one referring to redness in the calf and one with a prohibition sign over a saltshaker) got CVI <0.75 (Table 1).

**Table 1:** Content validity index concerning the first assessment round by experts

Illustration	Content validity index		
	Clarity	Theoretical relevance	Practical relevance
1	0.83	1.00	1.00
2	0.83	1.00	1.00
3	0.67	0.83	0.67
4	0.83	0.83	0.83
5	0.83	0.83	0.83
6	1.00	1.00	1.00
7	0.83	0.67	0.67
8	0.83	0.83	0.83
9	0.83	0.83	0.83

The experts considered the illustration referring to redness in the calf was not very clear and not very relevant, since the place highlighted in red and purple resembled the image of a venous ulcer. The image was therefore revised, and a larger area of the lower limb was highlighted in red only.

The illustration of a prohibition symbol over a saltshaker was considered of little relevance and of little theoretical relevance by the experts. They considered the information provided by the illustration was vague and had no specific relationship with VTE. The experts suggested replacing the illustration by a graphical representation of exercises for the lower limbs, which can be performed when the patient remains seated for more than 2 hours.

In addition, the experts made suggestions for improving the distribution of illustrations and text on the first page of the manual, to reduce visual pollution. They also replaced the term “arterial hypertension” by “high blood pressure”, also suggesting a better description of the term “long trips”.

The suggested changes were made, including new formatting by a second graphic designer. The manual was sent back to the same group of experts for a new round of assessment. The questionnaire should be returned electronically within 15 days and, when not returned, within 10 additional days. In the second round, 100% of experts responded, and all text items and illustrations had their content validated, with CVI ranging from 0.83 to 1.00.

For face validation, 42 lay people were invited to participate, and 40 (95.2%) accepted the invitation. The mean age of the sample was 42.8±18.9 years (minimum 18 and maximum 91 years). Of these, 21 (52.5%) were male, and most had complete or incomplete secondary education (53.0%), followed by 27.0% with complete or incomplete primary education, and 20.0% with undergraduate or graduate education.

Table 2 depicts the qualities desired for an informative manual on VTE prevention assessed by the lay population and the respective response categories.

**Table 2:** Features of the informative manual on prevention of thromboembolism as assessed by lay people and response percentages

Questions	Yes n (%)	No n (%)
Is the type of paper adequate?	40 (100.0)	-
Is the font size adequate?	40 (100.0)	-
Is the font readable?	40 (100.0)	-
Were you able to understand the information found on the manual?	39 (97.5)	1 (2.5)
Is the manual esthetically beautiful?	38 (95.0)	2 (5.0)
Is the manual reading tiresome?	4 (10.0)	36 (90.0)

Everyone considered adequate the paper type, font size and readability. Four people (10.0%) considered reading the manual tiresome, however did not suggest any improvement. The manual was considered valid for the target public.

**Figure 1:**
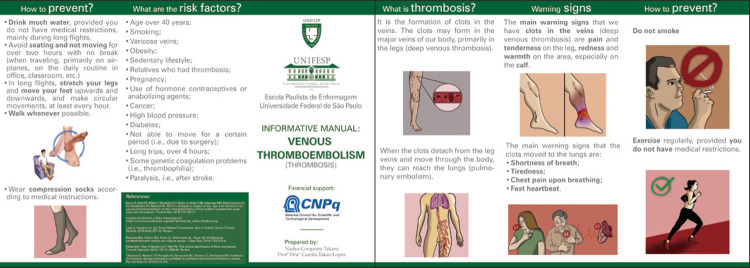
Final version of the manual on venous thromboembolism prevention for lay people

Figure 1 presents the version of the VTE prevention manual for lay people after content and face validation.

## DISCUSSION

Venous thromboembolism is one of the most frequent vascular diseases in the outpatient or inpatient population, and in those traveling long distances.^(26)^ Although 50.0% of thromboembolic events are related to current or recent hospitalization,^(27)^ preventive education on the disease can be used in any healthcare setting.

An American study quantified the level of knowledge about VTE of 325 people in the general public. Among lay participants, only 30.0% knew about DVT and PTE, 54.0% were not sure about the signs of DVT, 50.0% were not sure about the signs of PTE, and 38.0% were not sure about risk factors for VTE or risk reduction measures. Among the participants familiar with the disease, only 27.0% had been oriented by a healthcare professional, but 87.0% preferred to obtain health-related information from these professionals. On the other hand, 80.0% of participants who were healthcare professionals indicated knowledge about DVT and PTE, 85.0% identified one or more risk factors, and 97.0% identified more than half of the risk reduction measures. This evidence suggests that nurses and other healthcare professionals are well informed about the subject, but information has not been transmitted to the general public.^(28)^

Similarly, a study conducted by IBOPE found that, although 56.0% of 1,008 participants “had heard” about VTE, 43.0% of them were unaware of preventive measures, 57.0% were unaware of the symptoms and consequences of the disease, and 64.0% of people at high risk for VTE did not know whether they were at risk of developing the disease.^(11)^

In this context, it is relevant for the lay population to be educated by healthcare professionals about measures for disease prevention. The use of an education manual based on scientific evidence enables the dissemination of knowledge in a uniform and easily understood language.^(16)^

In this study, an informative manual on VTE prevention was developed. Adequate evidences of content validity was obtained through the opinion of experts, and satisfactory evidence of face validity was obtained from the opinion of the target population.

One of the most used techniques for the validation of information manuals in nursing is the Delphi technique, which seeks consensus on the opinion of specialists, through a systematic judgment. In 2013, a review study identified articles that used the technique and, in 67.0% of articles, two rounds of judgment were necessary to reach consensus among experts.^(29)^ This result corroborates the findings of this study.

The creation of an informative manual on VTE for the lay population enables a communication channel between nurses and the community, clarifies potential doubts, and provides knowledge about the disease, warning signs and how to recognize them, risk factors and preventive actions, in addition to contributing to the dissemination of knowledge to the population. Subjects can choose the level and amount of information that best suit them.^(13)^ The manual can be used to assist the verbal education of hospitalized people, in an outpatient context or in the context of Primary Care.

However, some difficulties in the process of preparing the manual were identified and are similar to those reported in other studies. Among them, language adequacy stands out, one of the main challenges for nurses when it comes to preparation of health-related education materials.^(30)^ Adjusting the language to the public in written education manuals enables providing the desired information and assists in the learning process of individuals.^(18)^

In view of the results obtained, we recognize that the convenience sample may have affected the generalization of the data, since the sample was representative of lay people with a higher level of education, not corresponding to the distribution of the Brazilian population. In addition, new recommendations on VTE prevention techniques have been published, suggesting periodic update of the manual content.

Further studies are needed to ascertain whether the use of the manual is associated with increased knowledge about VTE prevention, and if increased knowledge, in turn, is associated with disease prevention by lay people.

## CONCLUSION

The informative manual on venous thromboembolism was prepared and considered valid by expert nurses and lay people. We believe that the manual can be used as education strategy for the population at large, to enhance knowledge about prevention of venous thromboembolism.
