# Supplementary material for: Development and validation of an informative manual on venous thromboembolism for the lay population
Source: Einstein (Sao Paulo). 2020 Sep 9;18:eAO5425. doi: 10.31744/einstein_journal/2020AO5425 (PMC7480492; doi:10.31744/einstein_journal/2020AO5425)
Supplement: Translation

# Translation: Elaboração e validação de manual informativo sobre tromboembolismo venoso para leigos

**Authors:** Nadya Cerqueira Takara, Natany da Costa Ferreira, Beatriz Murata Murakami, Camila Takao Lopes

**Affiliations:** 1 Brasil Universidade Federal de São Paulo, São Paulo, SP, Brasil.; 2 Brasil Universidade de São Paulo, São Paulo, SP, Brasil.; 3 Brasil Hospital Israelita Albert Einstein, São Paulo, SP, Brasil.

**Keywords:** Tromboembolia venosa, Fatores de risco, Educação em enfermagem, Embolia pulmonar, Estudo de validação

## Abstract

**Objetivo:**

Elaborar um manual informativo sobre prevenção de tromboembolismo venoso para população leiga e estimar evidências de validade de conteúdo e de face.

**Métodos:**

Estudo metodológico realizado em três etapas. A primeira etapa foi a construção do manual; a segunda foi a validação de conteúdo com especialistas na área cardiovascular, que julgaram clareza, relevância teórica e pertinência prática, em uma escala tipo Likert de 4 pontos. Itens com índice de validade de conteúdo ≤0,75 foram revisados e reavaliados. A última etapa foi a validação de face por pessoas leigas, entrevistadas quanto à compreensão dos itens e à aparência visual. Itens com opiniões positivas por mais de 80,0% foram considerados adequados.

**Resultados:**

O manual foi elaborado contendo nove imagens, com definição da doença, fatores de risco, sinais e sintomas e medidas preventivas. Na primeira rodada de avaliação, os índices de validade foram de 1,0 para o texto de todas as seções, com sugestões de ajustes de linguagem. Em relação às imagens, os índices de validade variaram de 0,67 a 1,0. Na segunda rodada, o índice de validade atingiu 1,0 para todos os itens. Na validação de face, 40 pessoas leigas participaram, e todas consideraram o tipo de papel e o tamanho da letra adequados, além da letra legível; 97,5% conseguiram entender as informações contidas no manual; 98,0% o consideraram esteticamente bonito; e 90,0% consideraram a leitura não cansativa.

**Conclusão:**

O manual sobre prevenção de tromboembolismo venoso foi elaborado, com conteúdo validado por especialistas e considerado adequado pela população leiga. Esses resultados sugerem que o manual pode ser empregado como estratégia educacional preventiva do tromboembolismo venoso.

## INTRODUÇÃO

O tromboembolismo venoso (TEV) inclui a trombose venosa profunda (TVP) e o tromboembolismo pulmonar (TEP) e é a terceira principal doença cardiovascular (DCV). A doença é caracterizada pela formação de trombos em veias profundas, principalmente nos membros inferiores, causando dores, edema, calor, sensibilidade e úlceras. Esse trombo pode se desprender das paredes dos vasos e percorrer toda a circulação, obstruindo os vasos pulmonares.^(1-4)^

No mundo, aproximadamente 10 milhões de pessoas são acometidas pela doença.^(2)^ Anualmente, mais de 500 mil de mortes na Europa e mais de 300 mil nos Estados Unidos ocorrem devido à TEV.^(5)^ Embora se mantenha alta, no Brasil, a taxa bruta de mortalidade por TEP diminuiu em 31%, de maneira progressiva e constante, de 2,8/100 mil em 1989 para 2,62/100 mil em 2010.^(6)^

De julho de 2018 a julho de 2019, o Sistema Único de Saúde (SUS) gastou cerca de R$ 29 milhões com mais de 47 mil internações por TEV, com taxa de mortalidade de 2,46%.^(7)^ Estes custos se devem ao tratamento do TEV, incluindo prevenção secundária crônica por 3 a 6 meses: anticoagulação com heparina não fracionada, heparina de baixo peso molecular, novos anticoagulantes orais e antagonistas da vitamina K.^(8)^ Para pacientes com dois ou mais casos de episódios trombóticos espontâneos, a terapia anticoagulante deve ser realizada por tempo indeterminado.^(9)^

Apesar da alta taxa de mortalidade associada à doença, o conhecimento público a respeito da TEV é baixo, comparado ao conhecimento sobre sintomas e fatores de risco de outras DCV.^(10)^ Conforme apontado por pesquisa do Instituto Brasileiro de Opinião Pública e Estatística (IBOPE) em 2010, a população leiga brasileira conhece a existência dessa doença, mas não sabe como tratá-la, preveni-la e nem controlá-la.^(11)^

Os fatores de risco para TEV incluem idade, sexo, etnia, índice de massa corporal/obesidade, uso de contraceptivos orais ou terapia hormonal, uso de corticosteroides, sedentarismo, câncer, cirurgias, traumas/fraturas, imobilização, gravidez/puerpério, viagens longas e infecções agudas, dentre outros.^(12)^ O fato de a população leiga desconhecer tais fatores de risco, bem como suas medidas preventivas, é um dificultador para a prevenção. Assim, estratégias educativas para a população leiga devem ser elaboradas, a fim de se evitarem o desenvolvimento da doença e as hospitalizações, e diminuir os custos associados ao tratamento. Os materiais escritos ajudam o paciente a se lembrar dos cuidados que deve realizar, uma vez que as informações verbais são muito densas, além de poderem ser consultados mesmo no momento em que o profissional não está presente.^(13)^

Um aspecto a ser considerado na utilização de material educativo impresso é a validade de tais instrumentos, a qual se refere à confirmação de que o uso deste condiz com o propósito de sua formulação.^(14)^ Estudos anteriores elaboraram e validaram materiais educativos escritos para orientação de pacientes em diferentes contextos.

Cruz et al.,^(15)^ construíram um manual para pacientes com câncer de cabeça e pescoço submetidos à radioterapia, após levantamento bibliográfico de artigos científicos e livros técnicos, e validaram o conteúdo por meio da opinião de profissionais especialistas.^(15)^ Outros estudos também consideraram a opinião do público-alvo dos materiais educativos. Lopes et al.,^(16)^ desenvolveram um manual informativo sobre banho no leito para pacientes coronarianos, posteriormente validado tanto por enfermeiros quanto por pacientes.^(16)^ O mesmo grupo, em seguida, elaborou um manual informativo sobre cateterismo cardíaco, também validado por ambos os grupos.^(17)^ Oliveira et al.,^(18)^ elaboraram uma cartilha sobre alimentação saudável para gestantes, a qual foi validada por profissionais especialistas e mulheres grávidas.^(18)^

Até onde sabemos, não foram elaborados e validados materiais educativos escritos para orientação de pessoas leigas quanto à prevenção de TEV. O desenvolvimento de tais materiais preencherá esta lacuna no conhecimento.

## OBJETIVO

Elaborar um manual informativo sobre prevenção de tromboembolismo venoso para a população leiga e estimar evidências de validade de conteúdo e de face.

## MÉTODOS

Estudo metodológico, realizado em três etapas: elaboração do manual, validação de conteúdo por juízes especialistas e validação de face por público leigo.

### Primeira etapa: elaboração do manual

O manual foi elaborado em 2018, por uma estudante do terceiro ano do curso de bacharelado em enfermagem, sob supervisão de uma docente, enfermeira, especialista em cardiologia, com título de doutorado.

Os textos foram baseados em artigos científicos publicados no portal de dados PubMed^®^ e na *Scientific Electronic Library Online* (SciELO), de 2014 a 2018.^(19-23)^ A busca, não sistematizada, foi realizada com os termos “tromboembolismo venoso” e “*venous thromboembolism*”, nos campos “título ou resumo”. Foram selecionadas pesquisas que abordassem a definição da doença, sinais e sintomas, fatores de risco e medidas preventivas.

As informações foram extraídas das pesquisas pela estudante e revisadas pela docente, tendo sido dispostas na versão inicial do manual com vistas a atender pessoas leigas alfabetizadas, desde o Ensino Fundamental incompleto. O manual foi elaborado em folha sulfite A4 de 90g/mm. As ilustrações e o formato de *folder* foram realizados por um designer gráfico.

A construção seguiu as seguintes recomendações de Hoffman et al.,^(13)^ para elaboração de materiais educacionais escritos efetivos em saúde: envolver os principais interessados, incluindo os pacientes; indicar claramente a finalidade do material; concentrar-se em fornecer informações focadas no comportamento (por exemplo: “é importante que você faça os exercícios todos os dias”); assegurar-se de que o conteúdo seja preciso, atualizado, com base em evidências e com fontes adequadamente referenciadas; incluir os nomes dos autores e ano de publicação no material; evitar linguagem julgadora ou paternalista; considerar um nível de leitura da quinta à sexta série; usar frases curtas, expressando apenas uma ideia por frase; usar palavras comuns, sempre que possível; evitar o uso de jargões ou abreviações; escrever na voz ativa e no estilo conversacional; escrever na segunda pessoa (por exemplo: “você” em vez de “o paciente”); sequenciar as informações para que as de maior interesse do paciente sejam apresentadas no início do material; usar subtítulos; apresentar as informações usando listas com tópicos sempre que possível; usar um tamanho mínimo de fonte de 12 pontos; evitar o uso de itálico e todas as letras maiúsculas; usar negrito apenas para enfatizar palavras-chave ou frases; garantir um bom contraste entre a cor da fonte (por exemplo: preto) e o fundo (por exemplo: branco); usar ilustrações apenas se melhorarem a compreensão do leitor; usar desenhos simples, que provavelmente sejam familiares ao leitor.

### Segunda etapa: validação de conteúdo

O manual informativo foi avaliado por uma amostra não probabilística, selecionada por conveniência, composta por enfermeiros especialistas na área cardiovascular, com experiência clínica mínima de 2 anos, acrescida de experiência nas áreas de ensino e de pesquisa, participantes de um grupo de estudos, pesquisa e assistência de uma universidade federal.

O tamanho da amostra foi determinado de acordo com as recomendações de Cassepp-Borges et al.,^(14)^ considerando seis especialistas como número mínimo aceitável para esse tipo de estudo. Os juízes especialistas foram convidados a participarem da pesquisa por correio eletrônico. Aqueles que aceitaram participar receberam um questionário para caracterização demográfica e profissional, um para avaliação do conteúdo do manual e o Termo de Consentimento Livre e Esclarecido (TCLE). As variáveis de caracterização dos juízes especialistas foram: sexo, idade, maior nível educacional, tempo de experiência clínica, no ensino e na pesquisa na área cardiovascular.

Os especialistas analisaram cada item do manual individualmente, utilizando os seguintes critérios psicométricos:^(24,25)^ clareza (o item foi redigido de forma que o conceito fosse compreensível e expressasse adequadamente o que se esperava medir); relevância teórica (o item refletiu os conceitos envolvidos, era relevante e adequado para atingir os objetivos propostos); pertinência prática (o item refletiu os conceitos envolvidos, era pertinente e adequado para atingir os objetivos propostos). As magnitudes de resposta, para todos os critérios, foram 1 para não claro/relevante/pertinente, 2 para pouco claro/relevante/pertinente, 3 para bastante claro/relevante/pertinente, 4 para muito claro/relevante/pertinente.

Além dos critérios psicométricos, foram solicitadas sugestões para aprimoramento do manual quando a avaliação fosse diferente de “4”. Os especialistas tiveram um prazo de 15 dias para devolução eletrônica dos questionários preenchidos. Em caso de não cumprimento do prazo de devolução inicial, novo contato eletrônico foi realizado, conferindo mais 10 dias para a devolução.

O índice de validação de conteúdo (IVC), que é a proporção de itens que recebe pontuação de 3 ou 4 pelos especialistas, foi calculado para cada item. Itens com IVC ≤0,75 foram reenviados aos especialistas para nova avaliação, até que o IVC aceitável fosse atingido.^(24,25)^

Os dados foram armazenados, organizados e analisados em planilha do software Microsoft Office Excel. As variáveis de caracterização dos especialistas foram descritas por medidas de tendência central e dispersão. Para as variáveis categóricas, foram calculadas as frequências absoluta e relativa; para as variáveis quantitativas, foram calculados média, desvio padrão, valores mínimo e máximo.

### Terceira etapa: validação de face

O manual informativo foi julgado por uma amostra não probabilística, selecionada por conveniência, composta por pessoas sem qualquer experiência profissional na área de saúde, com idade maior ou igual a 18 anos, de ambos os sexos.

O recrutamento foi realizado pela estudante em um condomínio residencial, quando foram explicados os objetivos do estudo e a forma de participação dos sujeitos. Aqueles que aceitaram participar assinaram o TCLE, leram o manual e responderam a um questionário sobre características demográficas. As variáveis de caracterização foram idade, sexo e nível de escolaridade.

Após o preenchimento de dados demográficos, o instrumento de avaliação do manual foi aplicado por meio de entrevista estruturada, com questões dicotômicas, conduzida pela estudante que elaborou o manual. Foram avaliadas a compreensibilidade das informações do manual, a carga de informações e os aspectos estéticos, como adequação do tamanho, tipo da fonte e tipo de papel utilizado para impressão. Itens com opiniões positivas por uma proporção igual ou maior a 80% da amostra foram considerados adequados.^(24,25)^

O projeto foi submetido e aprovado pelo Comitê de Ética em Pesquisa da Universidade Federal de São Paulo (CAAE: 93294718.2.0000.5505, protocolo 2.795.768). Aos juízes especialistas e pessoas leigas, foram garantidos sigilo e anonimato.

## RESULTADOS

O manual foi inicialmente elaborado com as seções “O que é trombose?”, “Quais são os fatores de risco de trombose?”, “Sinais de alerta da trombose” e “Como prevenir a trombose?”.

Para a validação de conteúdo, dez especialistas foram convidados, e sete (70,0%) aceitaram participar do estudo. Foram devolvidos seis (85,7%) instrumentos preenchidos.

Todos os especialistas eram do sexo feminino (6; 100%) e tinham média de idade de 30,3 (±3,3) anos. A maioria tinha Mestrado (4; 66,6%); 6,3 (±2,4) anos de experiência clínica; 6,8 (±3,5) anos de experiência em ensino e 2,2 (±4,4) anos de experiência em pesquisa na área cardiovascular.

Na primeira rodada de validação de conteúdo, apenas duas ilustrações (uma referente à vermelhidão em panturrilha e uma com um símbolo de proibição sobre um saleiro) tiveram IVC <0,75 (Tabela 1).

**Tabela 1:** Índice de validade de conteúdo referente à primeira rodada de avaliação pelos juízes especialistas

Imagem	Índice de validade de conteúdo		
	Clareza	Relevância teórica	Pertinência prática
1	0,83	1,00	1,00
2	0,83	1,00	1,00
3	0,67	0,83	0,67
4	0,83	0,83	0,83
5	0,83	0,83	0,83
6	1,00	1,00	1,00
7	0,83	0,67	0,67
8	0,83	0,83	0,83
9	0,83	0,83	0,83

Os juízes consideraram que a ilustração referente à vermelhidão em panturrilha foi considerada pouco clara e pouco pertinente, pois o local destacado pelas cores vermelho e roxo lembrava a imagem de uma úlcera venosa. Dessa forma, a imagem foi revisada e destacou-se apenas em cor vermelha uma área mais abrangente do membro inferior.

A ilustração de um símbolo de proibição sobre um saleiro foi considerada pouco pertinente e com pouca relevância teórica para os juízes. Estes julgaram que as informações fornecidas pela imagem eram vagas e não tinham relação específica com TEV. Os juízes sugeriram a substituição da imagem por uma representação gráfica de exercícios para os membros inferiores que podem ser realizados quando o paciente permanecer sentado por tempo superior a 2 horas.

Além disso, os juízes fizeram sugestões para o aprimoramento da distribuição das imagens e texto na página inicial do manual, de forma a diminuir a poluição visual e também substituíram o termo “hipertensão arterial” por “pressão alta” e melhor descrição do termo “viagens longas”.

As modificações sugeridas foram realizadas, incluindo nova formatação por um segundo designer gráfico. O manual foi reenviado ao mesmo grupo de juízes para nova rodada de apreciação. Foram estabelecidos 15 dias para devolução eletrônica do questionário e, quando não devolvido, 10 dias adicionais. Na segunda rodada, houve resposta de 100% dos especialistas, e todos os itens textuais e de imagens tiveram o conteúdo validado, com IVC variando de 0,83 a 1,00.

Para validação de face, 42 pessoas leigas foram convidadas a participar, e 40 (95,2%) aceitaram o convite. A média da idade da amostra foi de 42,8±18,9 anos, (mínimo de 18 e máximo de 91). Destas, 21 (52,5%) eram do sexo masculino, e a maioria tinha Ensino Médio completo ou incompleto (53,0%), seguida de 27,0% com Ensino Fundamental completo ou incompleto e de 20,0% com Ensino Superior ou Pós-Graduação.

A tabela 2 apresenta as qualidades desejadas do manual informativo de prevenção de TEV avaliadas pelo público leigo e respectivas categorias de respostas.

**Tabela 2:** Qualidades do manual informativo de prevenção de tromboembolismo avaliadas por pessoas leigas e percentuais de respostas

Questões	Sim n (%)	Não n (%)
O tipo de papel está adequado?	40 (100,0)	-
O tamanho da letra está adequado?	40 (100,0)	-
A letra está legível?	40 (100,0)	-
Você conseguiu entender as informações contidas no manual?	39 (97,5)	1 (2,5)
O manual está esteticamente bonito?	38 (95,0)	2 (5,0)
A leitura do manual está cansativa?	4 (10,0)	36 (90,0)

Todas as pessoas consideraram o tipo de papel, o tamanho e a legibilidade da letra do manual educativo adequados. Quatro pessoas (10,0%) consideraram a leitura do manual cansativa, porém não souberam sugerir melhorias. O manual foi considerado válido para o público-alvo.

A figura 1 apresenta a versão do manual de prevenção de TEV para pessoas leigas após validação de conteúdo e de face.

**Figura 1:** Versão final do manual sobre prevenção de tromboembolismo venoso para pessoas leigas

## DISCUSSÃO

O TEV é uma das doenças vasculares mais frequentes na população ambulatorial, hospitalizada e que viaja longas distâncias.^(26)^ Embora 50,0% dos eventos tromboembólicos estejam relacionados à hospitalização atual ou recente,^(27)^ orientações preventivas da doença podem ser instituídas em qualquer cenário de saúde.

Estudo americano quantificou o nível de conhecimento de 325 pessoas do público em geral sobre TEV. Dentre os participantes leigos, apenas 30,0% conheciam TVP e TEP, 54,0% não tinham certeza a respeito dos sinais de TVP, 50,0% não tinham certeza a respeito dos sinais de TEP, e 38,0% não tinham certeza sobre os fatores de risco para TEV ou sobre medidas de redução de risco. Dentre os participantes familiarizados com a doença, apenas 27,0% tinham sido orientados por um profissional de saúde, porém 87,0% preferem obter informações de saúde a partir destes profissionais. Por outro lado, 80,0% dos participantes que eram profissionais da saúde indicaram conhecimento sobre TVP e TEP, 85,0% identificaram um ou mais fatores de risco, e 97,0% identificaram mais da metade das medidas de redução de risco. Estas evidências sugerem que os enfermeiros e demais profissionais de saúde são bem informados sobre o assunto, mas as informações não têm sido transmitidas ao público em geral.^(28)^

Similarmente, estudo realizado pelo IBOPE verificou que, embora 56,0% dos 1.008 participantes tenham “ouvido falar” sobre TEV, 43,0% destes desconheciam medidas preventivas, 57,0% desconheciam os sintomas e consequências da doença, e 64,0% das pessoas com alto risco para TEV não sabiam dizer se corriam risco de desenvolver a doença.^(11)^

Neste contexto, é relevante que a população leiga seja educada quanto às medidas preventivas da doença pelos profissionais da saúde. O emprego de um manual educativo baseado em evidências científicas possibilita a disseminação de conhecimento em uma linguagem uniforme e de fácil compreensão.^(16)^

Neste estudo, um manual informativo sobre prevenção de TEV foi elaborado. Adequadas evidências de validade de conteúdo foram obtidas, por meio da opinião de juízes especialistas, e evidências de validade de face satisfatórias foram obtidas a partir da opinião do público-alvo.

Uma das técnicas mais empregadas para validação de manuais informativos na enfermagem é a técnica Delphi, que busca o consenso da opinião de especialistas, por meio de um sistema de julgamento sistematizado. Em 2013, estudo de revisão identificou artigos que empregaram a técnica e, em 67,0% dos artigos, duas rodadas de julgamento foram necessárias para atingir o consenso entre os *experts*.^(29)^ Esse resultado corrobora achados deste estudo.

A criação de um manual informativo sobre TEV para a população leiga possibilita um canal de comunicação entre os enfermeiros e a comunidade, esclarece potenciais dúvidas, e proporciona o conhecimento acerca da doença, sinais de alerta e como reconhecê-los, fatores de risco e ações preventivas, além de contribuir para a disseminação do conhecimento para a população. O sujeito pode escolher o nível e a quantidade de informações que melhor o atendem.^(13)^ O manual pode ser utilizado para auxiliar a orientação verbal de pessoas hospitalizadas, em contexto ambulatorial ou no contexto da Atenção Primária.

Porém, algumas dificuldades no processo de elaboração do manual foram identificadas e são semelhantes às identificadas em outros estudos. Dentre elas, destacam-se a adequação da linguagem, um dos principais desafios dos enfermeiros no que tange à elaboração de materiais educacionais em saúde.^(30)^ A adaptação da linguagem ao público em manuais educativos escritos possibilita a transmissão das informações desejadas e auxilia no processo de aprendizado dos indivíduos.^(18)^

Diante dos resultados obtidos, reconhece-se que a amostragem de conveniência pode ter afetado a generalização dos dados, visto que a amostra foi representativa de pessoas leigas com maior nível de escolaridade, não correspondendo à distribuição da população brasileira. Além disso, novas recomendações acerca de técnicas de prevenção do TEV vêm sendo publicadas, sugerindo a atualização do conteúdo do manual periodicamente.

Futuros estudos são necessários para averiguar se o uso do manual se associa ao aumento de conhecimento sobre prevenção de TEV e se o aumento do conhecimento, por sua vez, associa-se à prevenção da doença por pessoas leigas.

## CONCLUSÃO

O manual informativo sobre tromboembolismo venoso foi elaborado e considerado válido por enfermeiros especialistas e pessoas leigas. Acredita-se que o manual poderá ser utilizado como estratégia educacional para aumentar o conhecimento sobre prevenção de tromboembolismo venoso para a população em geral.
